# Influence of density‐dependent competition on foraging and migratory behavior of a subtropical colonial seabird

**DOI:** 10.1002/ece3.3216

**Published:** 2017-07-10

**Authors:** Juliet S. Lamb, Yvan G. Satgé, Patrick G. R. Jodice

**Affiliations:** ^1^ Department of Forestry and Environmental Conservation Clemson University Clemson SC USA; ^2^ South Carolina Cooperative Fish and Wildlife Research Unit Clemson SC USA; ^3^ U.S. Geological Survey South Carolina Cooperative Fish and Wildlife Research Unit Clemson SC USA

**Keywords:** Ashmole's halo, colony size, competition, migration, pelican

## Abstract

Density‐dependent competition for food resources influences both foraging ecology and reproduction in a variety of animals. The relationship between colony size, local prey depletion, and reproductive output in colonial central‐place foragers has been extensively studied in seabirds; however, most studies have focused on effects of intraspecific competition during the breeding season, while little is known about whether density‐dependent resource depletion influences individual migratory behavior outside the breeding season. Using breeding colony size as a surrogate for intraspecific resource competition, we tested for effects of colony size on breeding home range, nestling health, and migratory patterns of a nearshore colonial seabird, the brown pelican (*Pelecanus occidentalis*), originating from seven breeding colonies of varying sizes in the subtropical northern Gulf of Mexico. We found evidence for density‐dependent effects on foraging behavior during the breeding season, as individual foraging areas increased linearly with the number of breeding pairs per colony. Contrary to our predictions, however, nestlings from more numerous colonies with larger foraging ranges did not experience either decreased condition or increased stress. During nonbreeding, individuals from larger colonies were more likely to migrate, and traveled longer distances, than individuals from smaller colonies, indicating that the influence of density‐dependent effects on distribution persists into the nonbreeding period. We also found significant effects of individual physical condition, particularly body size, on migratory behavior, which in combination with colony size suggesting that dominant individuals remain closer to breeding sites during winter. We conclude that density‐dependent competition may be an important driver of both the extent of foraging ranges and the degree of migration exhibited by brown pelicans. However, the effects of density‐dependent competition on breeding success and population regulation remain uncertain in this system.

## 1 INTRODUCTION

Density dependence, or the feedback between population size and population growth rate, acts as a stabilizing mechanism in ecological communities by altering individual behavior and fitness (Fowler, 1987; Mylis & Diekmann, 1995). At low organism densities, effects of increasing population size on individual fitness are typically positive and may include increasing reproductive success and decreased mortality, a process known as the Allee Effect (Courchamp, Clutton‐Brock, & Grenfell, 1999). As organism densities continue to increase, however, conspecific competition and interference can act to reduce individual vital rates until recruitment and mortality reach equilibrium (Brook & Bradshaw, 2006). One of the principal mechanisms underlying the shift from positive to negative density‐dependent effects is the increase in intraspecific competition coincident with increasing population size, which can result in lower resource availability and reduced individual fitness (Fowler, 1987).

Colonial animals provide a unique model to assess density‐dependent mechanisms, as they selectively congregate in areas of locally high population densities, and the number of colony members (hereafter, colony size) can vary widely within a species and ecosystems. Individuals living in colonies experience both costs and benefits of colony membership that can be mediated in part by colony size; therefore, the optimal size of a colony is one that maximizes lifetime reproductive success for individual colony members by providing the largest possible ratio of benefits to costs (Brown & Orians, 1970; Brown, Stutchbury, & Walsh, 1990). For colonial breeders, individual foraging ranges during the breeding period are often constrained by the need to return to the colony site at regular intervals to feed offspring, a process known as central‐place foraging (Orians & Pearson, 1979). Thus, both foraging effort and offspring condition are frequently used to test for the presence and direction of density‐dependent effects in colonial breeders. For example, a positive relationship between foraging success and colony size could result from the use of social information to locate and harvest food resources more quickly and efficiently (Brown & Brown, 1996; Donaldson‐Matasci, DeGrandi‐Hoffman, & Dornhaus, 2013). Alternatively, because the foraging ranges of central‐place foragers are limited at some level by energetic constraints (Orians & Pearson, 1979), individuals in more numerous colonies experience intensified localized competition for food resources, which results in reduced foraging success or increased foraging costs due to direct resource depletion, conspecific interference, and altered prey behavior (Kuhn, Baker, Towell, & Ream, 2014; Lewis, Sherratt, Hamer, & Wanless, 2001).

Colonially‐breeding seabirds have frequently been a model system for studying the factors that regulate colony size (Coulson, 2002). Density‐dependent reduction in resource availability around seabird colony sites relative to colony size is commonly referred to as Ashmole's halo (Birt, Birt, Goulet, Cairns, & Montevecchi, 1987; Gaston, Ydenberg, & Smith, 2007; Hemerik, Van Opheusden, & Ydenberg, 2014). As Ashmole (1963) first proposed density‐dependent resource depletion as a stabilizing mechanism for colony size in seabirds, extensive research has focused on testing this hypothesis across seabirds and other avian taxa. In addition to directly measuring reduced prey abundance around colony sites (Bonal & Aparicio, 2008), previous work has suggested that higher numbers of conspecifics can lead to proportional reductions in individual foraging efficiency (Møller, 1987), and reproductive output (Hoi, Hoi, Kristofik, & Darolova, 2006). Adult foraging effort, as measured by distance or duration of foraging movements (e.g., Ainley et al., 2004; Ballance, Ainley, Ballard, & Barton, 2009; Ford, Ainley, Brown, Suryan, & Irons, 2007), is a common metric used to assess density‐dependent competition particularly in marine systems where measuring prey availability over expansive areas can be prohibitive. Seabirds are expected to respond to density‐dependent reduction in prey availability by increasing their foraging distances, subsequently reducing feeding rates to nestlings, and ultimately decreasing nestling condition and survival (e.g., Gaston, Chapdelaine, & Noble, 1983; Hunt, Eppley, & Schneider, 1986; Tella et al., 2001).

The majority of studies assessing density dependence in relation to size of seabird colonies have focused on the breeding season, during which individual movements are limited by central‐place foraging restrictions. However, on a broader scale, seasonal migration also represents a central‐place behavior, in that migratory animals, particularly those with high breeding site fidelity, are constrained by the need to return to their breeding sites in subsequent seasons (Hoover, 2003; Naves, Monnat, & Cam, 2006). In tropical regions, seabirds frequently display partial migration (Lack, 1944), in which migration distances vary widely between individuals and some breeders do not migrate (Lundberg, 1988). If remaining closer to the breeding site during winter represents an energetic or competitive advantage (e.g., Chapman, Brönmark, Nilsson, & Hansson, 2011; Pérez, Granadeiro, Dias, Alonso, & Catry, 2013), it is possible that density‐dependent competition for limited resources could also act on migratory behavior. To date, there are few examples of studies assessing the relationship between colony size and migration. Diamond (1978) tested colony size relationships across several tropical species and found that species that bred in larger colonies were more likely to migrate than species with smaller average colony sizes. This remains the only example of density‐dependent constraints on migratory patterns in seabirds, and it focused on species‐wide patterns rather than individual strategies.

We investigated the relationship between colony size, breeding home range, nestling condition, and migration patterns in the Eastern brown pelican (*Pelecanus occidentalis carolinensis*) in the northern Gulf of Mexico. Brown pelicans are among the largest‐bodied seabirds, meaning that both interspecific competition and predation are limited and prey availability is likely the principal driver of breeding success. Moreover, brown pelicans are partially migratory in this portion of their range (King et al., 2013), making them a useful species for testing for effects of density‐dependent competition on migration. We collected year‐round GPS locations of nesting adults from breeding colonies of various sizes, as well as measurements of chick condition, to test the influence of colony size on movement and reproductive parameters. Based on Ashmole's hypotheses, we predicted that, after controlling for factors such as individual physical characteristics and environmental conditions, pelicans nesting in breeding colonies with greater numbers of conspecifics would (1) travel greater distances to forage during breeding; (2) raise poorer‐quality nestlings; and (3) be more likely to migrate, and winter farther from their breeding sites, than those nesting at smaller colonies.

## 2 MATERIALS AND METHODS

### 2.1 Focal species and study area

**Figure 1:**
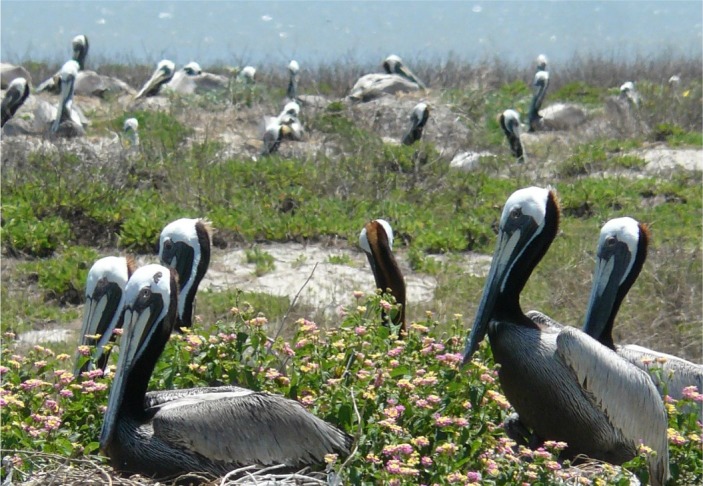
Brown pelican colony at Chester Island, Texas (J. Lamb)

The Eastern brown pelican (Figure 1) is a large‐bodied seabird that nests in colonies of 10 to upwards of 5,000 pairs, on nearshore barrier islands in subtropical and tropical North American waters. It breeds between March and August, laying 2–3 eggs and raising 1–2 chicks per year. The species is facultatively migratory during nonbreeding, with some individuals remaining resident and others leaving breeding areas (King et al., 2013). Pelicans forage in near‐ and offshore waters and capture schooling fish by plunge‐diving.

**Figure 2:**
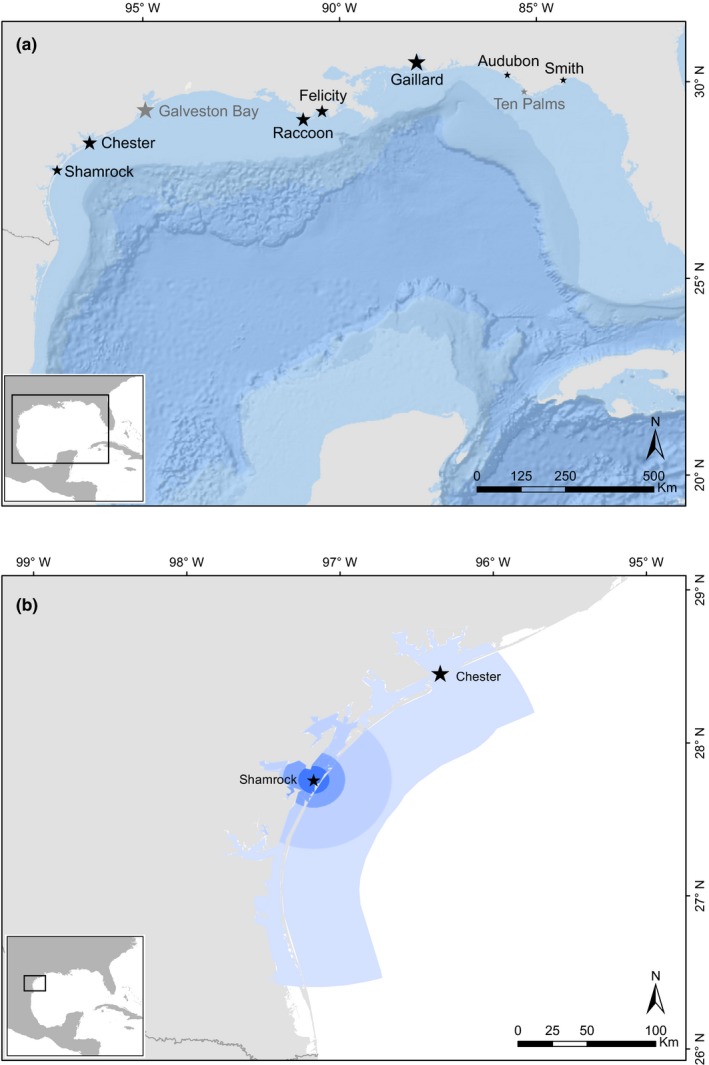
(a) Brown pelican study colonies in the Gulf of Mexico. Size of star indicates relative size of colony. Darker colonies were sampled for both adult movements and netling health; lighter colonies for nestling health only. (b) An example of buffer widths used to calculate local environmental conditions for each colony. Environmental variables were averaged over 10, 20, 50, and 150 km radii (colored from darkest to lightest), bounded by a 50‐m distance from the shoreline

We collected data on breeding adult movements and nestling health of pelicans at seven colonies in the Northern Gulf of Mexico between 83° and 98° W and 27° and 31° N (Figure 2a). Two additional colonies were sampled for nestling health, but not for adult movements. All colony sites were within the same marine ecoregion (Spalding et al., 2007). The number of breeding pairs at each study site was obtained from the most recent colonial waterbird census data collected (Table 1).

**Table 1:** Colony characteristics and measurements of tracked adults captured at six brown pelican breeding colonies in the northern Gulf of Mexico, 2013–2014. Mean values are reported, with *SD*

	Smith	Audubon	Gaillard	Felicity	Raccoon	Shamrock	Chester
Colony size (breeding pairs)	40^a^	100^a^	4500^b^	1800^c^	4300^c^	1400^d^	3200^d^
# of adults tracked	9	11	5	12	14	11	10
% male	78	64	40	50	57	55	30
Mass (g)	3414 ± 432	3414 ± 558	3190 ± 329	3448 ± 36	3546 ± 353	3459 ± 562	3070 ± 508
Culmen length (mm)	322 ± 22	315 ± 21	312 ± 20	313 ± 23	316 ± 23	321 ± 25	309 ± 19
BCI^e^	−141 ± 273	−241 ± 205	−131 ± 343	77 ± 195	121 ± 263	−19 ± 306	−147 ± 281

Data sources:

a Florida Fish and Wildlife Conservation Commission (unpublished).

b Dauphin Island Sea Labs (unpublished).

c Walter, Leberg, Dindo, and Karubian (2014).

d Texas Colonial Waterbird Census (unpublished).

e Body Condition Index (BCI) is a derived parameter representing the relationship between mass and skeletal size. Positive values indicate higher mass than predicted by the regression between mass and skeletal size, while negative values indicate lower mass than predicted.

Islands supporting pelican colonies also contained a mixture of other nesting species, principally herons and egrets (Ardeidae), black skimmers (*Rynchops niger*), terns (Sternidae), and laughing gulls (*Leucophaeus atricilla*). For the purposes of this study, we included only the number of conspecifics present at a colony (intraspecific competition) rather than the overall number of nesting birds present (interspecific competition). The other species that share pelican breeding and foraging areas use different foraging habitats, employ different feeding strategies and target different sizes and species of prey than do brown pelicans (De Graaf, Tilghman, & Anderson, 1985), meaning that effects of interspecific competition on distribution and behavior of brown pelican prey are likely to be minimal. Laughing gulls, which are kleptoparasitic feeders and may target brown pelicans, were not accurately censused in the study region; however, our observations suggest they were present at similar densities across all study colonies.

### 2.2 Environmental characteristics

As fine‐scale data on forage fish concentrations are not available for the study area, we used remotely sensed environmental parameters to compare the marine environments surrounding each study colony and account for potential variation in prey availability between colony sites. We selected environmental parameters related to preferred habitats of small schooling fish (Christmas, McBee, Waller, & Sutter, 1982), particularly Gulf menhaden, which are considered a principal prey resource for brown pelicans (Ahrenholz, 1991; Fogarty, Nesbitt, & Gilbert, 1981). We included five remotely‐sensed environmental variables (Table S1) measured at distances of 10, 20, 50, and 150 km from the colony, bounded by the coastline and up to 50 km offshore (Figure 2b). Two parameters were fixed (bathymetry, bottom substrate: Harris, Macmillan‐Lawler, Rupp, & Baker, 2014), and three were variable (salinity, sea surface temperature: Boyer et al., 2011; net primary production: Behrenfeld & Falkowski, 1997). We averaged variable parameters within four seasons per year: January–March (winter); April–June (spring); July–September (summer); and October–December (fall). We conducted a principal components analysis (PCA) using the “vegan” package (Oksanen, 2016) in R (R Core Team, 2014) to collapse environmental variables into composite discriminant axes.

### 2.3 Adult tracking

To assess foraging ranges of adult pelicans, we deployed 65 g solar GPS Platform Terminal transmitters (GeoTrak, Inc., North Carolina, USA) with a backpack‐style Teflon ribbon harness attachment (Dunstan, 1972). To elevate the transmitters and prevent feathers from covering the solar panels and antenna, we mounted each device on a 6‐mm‐thick neoprene pad that also extended 6 mm beyond the perimeter of the transmitter in all directions. Transmitters were programmed to collect 12 fixes/day during breeding (April–August; every 90 min from 1030 to 0130 GMT), 10 fixes/day during pre‐ and postbreeding (September–October and February–March; every 90 min from 0700 to 0100 GMT), and 8 fixes/day during winter (November–January; every 120 min from 0700 to 0100 GMT). We obtained an average error estimate for GPS points from transmitters at known locations (*N* = 220) of 4.03 ± 2.79 m. Adults were captured at active nests using leg nooses in either the late incubation or early chick‐rearing stage of breeding. Nest contents were recorded, including number and age of chicks present and number and status of eggs present. All captured adults were weighed, measured, banded, and sampled for blood and feathers. As morphology is not always sufficient to determine sex in brown pelicans, adults were later sexed via PCR using collected DNA samples (Itoh et al., 2001). Total handling time from capture to release averaged 19 min (±6.5 min).

To calculate adult physical condition, we followed previous literature in assuming a linear relationship between culmen length (as an index of skeletal size) and body mass (Eggert, Jodice, & O'Reilly, 2010). We used the best‐fitting regression equation to calculate the difference between each individual's measured body mass and its predicted body mass based on skeletal size, which we considered its body condition index (BCI). Thus, a negative BCI indicates an individual in poorer‐than‐average physical condition, while a positive BCI indicated better‐than‐average physical condition. As brown pelicans are sexually dimorphic (Shields, 2014), we calculated BCI separately by sex to account for bimodal distribution of body size and ensure that assumptions of normality were met.

### 2.4 Breeding‐season home ranges

Given the high resolution of GPS data, we were able to infer nest attendance from subsequent locations of adults, and thus, we considered all data points collected between transmitter attachment and the date that the adult discontinued regular nest attendance as breeding‐season movements. For adults that remained resident on the colony after the breeding period had ended, we imposed a cutoff for breeding‐season movements at 90 days after inferred hatch date, which represents the maximum recorded fledging period in this species (Shields, 2014). Although GPS tags collected data over multiple years for some individuals, we included only the first year of data for each individual to maximize sample size and improve comparisons among individuals. GPS data were visually assessed and outliers (i.e., points that required flight speeds in excess of 65 km per hour: Schnell & Hellack, 1978) manually removed. We determined 50% and 95% kernel density home ranges for each individual using the “ks” package in R (Duong, 2015) with a plugin bandwidth estimator (Gitzen, Millspaugh, & Kernohan, 2006; Wand & Jones, 1994). Finally, we calculated the areas included within the 50% utilization distributions (50UD: core) and 95% utilization distributions (95UD: full) home range contours using the “rgeos” package in R (Bivand & Rundel, 2017) with Albers Conic Equal‐area projections centered on each region.

### 2.5 Chick condition and stress

Between 2013 and 2015, we sampled a subset of nestlings at each of the seven sites where adult pelicans were tracked, as well as two additional colony sites (Figure 2a). We captured nestlings at 3–4 weeks of age (25 June ± 13 days) and measured the mass, culmen length, tarsus, and wing lengths, as well as collecting a sample of body feathers from each nestling. We normalized culmen, tarsus, and wing length measurements and conducted a principal components analysis (PCA) to generate a composite measure of skeletal size (e.g., Benson, Suryan, & Piatt, 2003). Using the first‐axis PCA scores, we then regressed body mass (response) on the index of skeletal size (predictor) and calculated the regression equation that best represented the relationship between the two measures. We chose a second‐order polynomial regression to reflect the asymptotic pattern of chick growth during development. We calculated BCI in the same manner as for adult pelicans.

We also measured the stress hormone corticosterone in feathers of chicks to assess condition over the course of development. As corticosterone levels in nestling tissues reflect nutritional stress during the growth period (Will et al., 2014), this measurement provides an additional integrated index of overall nutritional conditions at a colony that might not be reflected by a one‐time measurement of chick body condition. We measured corticosterone levels in feathers using a radioimmunoassay procedure similar to the one developed by Bortolotti, Marchant, Blas, and German (2008). Briefly, we removed the feather rachis, cut each feather into small (<0.5 mm) segments, extracted corticosterone in three successive methanol washes, reconstituted samples in buffer, and measured corticosterone concentrations via radioimmunoassay (MP Biomedicals, California, USA). Complete details of corticosterone analysis are described in Lamb, O'Reilly, and Jodice (2016).

### 2.6 Migratory movements

To classify adults as migratory or nonmigratory, we defined winter home ranges as all points following the final postbreeding dispersal in fall/winter, preceding the return to the breeding colony the following spring. Using only these locations, we approximated individual winter home ranges using 95% KDEs. If an individual's breeding‐season home range (95UD) overlapped its winter home range, we classified its migratory strategy as resident (Cagnacci et al., 2016); all remaining individuals were classified as migratory. Under this classification scheme, all individuals classified as migratory had summer and winter home ranges separated by more than 100 km; therefore, we felt confident that classifications obtained using this method were biologically meaningful. However, reduced prey availability in winter might be expected to increase the foraging range of resident birds, meaning that the zone of prey depletion around a breeding colony might shift outside the boundaries of summer foraging ranges. We thus calculated migration distances as an additional measure of migratory behavior, using the linear distance between an individual's breeding colony and the centroid of the 95% KDE of its winter locations.

### 2.7 Statistical analyses

We modeled nestling health measurements (BCI and CORT) as a function of colony size environmental characteristics (principal component 1 and/or 2), and their interaction. We included year in models as a random factor. We modeled individual adult home ranges (50 UD, 95 UD) and migratory parameters (migration strategy, migration distance) using full‐factorial generalized linear models as a function of colony size, environmental characteristics (principal component 1 and/or 2), and individual characteristics (body size (culmen length), sex, and BCI). In all cases, the global model including all five predictor variables fit the data well (Hosmer–Lemeshow goodness‐of‐fit tests, *p* > .1 for all). We selected the best candidate models using Akaike's information criterion (AIC_c_) values. Models that increased AIC_c_ by ≤2 relative to the top model were substantially supported, while models with Δ AIC_c_ of 4–7 received weak support (Burnham & Anderson, 2004). We calculated means‐parameterized model‐averaged coefficients and importance values for each predictor based on the full 95% confidence set of tested models. We conducted model selection using the “AICcmodavg” package in R (Mazerolle, 2016). To assess relationships between individual predictor and response variables, we used univariate linear models.

## 3 RESULTS

### 3.1 Colony characteristics

Pelican colonies included in this study spanned the northern coast of the Gulf of Mexico and ranged in size from 75 to 5,000 breeding pairs (Figure 2a). The first two axes together explained 76% of the variance between colony sites. The first principal components axis of environmental characteristics explained 48% of intercolony variation. Colonies differed primarily in salinity parameters at all scales and bottom substrate (proportion of mud relative to sand) within 10 and 20 km of the colony site. On the second principal components axis, which explained 28% of variation, colonies differed primarily in net primary production and spring and summer sea surface temperatures at all scales. We used the scores of each colony on each of the first two axes to represent environmental characteristics in subsequent models.

### 3.2 Breeding‐season home ranges

**Figure 3:**
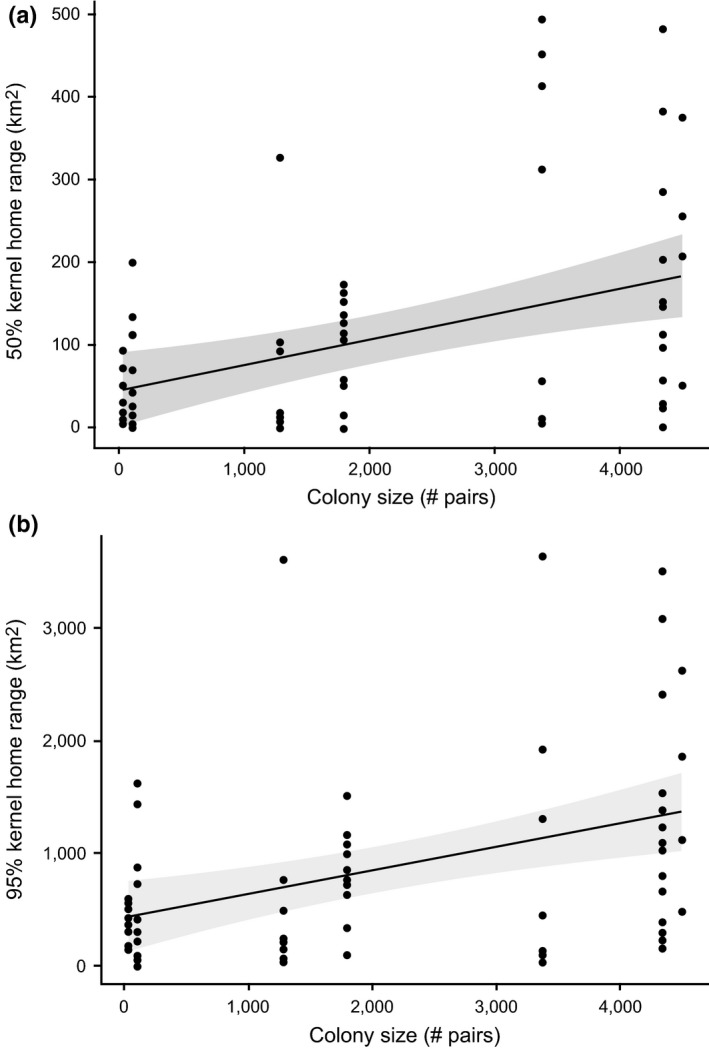
Mean home range areas [(a) 50% kernel; (b) 95% kernel] of breeding adult brown pelicans in the Gulf of Mexico, 2013–2015. Ninety five percent confidence estimates of regression lines are shaded

The number of birds captured at each colony ranged from 5 to 14 (μ = 10.3; Table 1). Colony size alone was the top predictor of individual 50UD and 95UD areas (Table 2). Overall, the linear relationship between colony size and breeding‐season home range size was significantly positive for both 50UD (*t*_65_ = 3.65, *p* = .005) and 95UD home ranges (*t*_65_ = 3.56, *p* = .007) (Table 3). For each increase of 100 breeding pairs at a colony, mean core home range size of individual breeders increased by approximately 3 km^2^ (Figure 3a) and mean full home range size increased by approximately 19 km^2^ (Figure 3b).

**Table 2:** Substantially supported (Δ AIC_c_ ≤ 2) generalized linear models, model weights (*w*_*i*_) and top model evidence ratios (*E*) for adult breeding and nonbreeding movements. Link functions are given in parentheses

	*K*	AIC_c_	_*i*_ (AIC_c_)	*w*_*i*_ (AIC_c_)	Σ*w*	*E*
**Breeding (** ***N*** **=** **73)**						
Core home range (gamma)						
Colony size	3	835.44	0	0.31	0.31	3.0
Full home range (gamma)						
Colony size	3	1094.34	0	0.27	0.27	2.12
Colony size + condition	4	1095.84	1.50	0.13	0.40	
**Nonbreeding (** ***N*** **=** **63)**						
Migratory strategy (binomial)						
Colony size + body size	4	80.82	0	0 .18	0.18	1.63
Colony size + body size + sex	5	81.80	0.97	0.11	0.29	
Colony size + body size + condition	5	81.87	1.05	0.11	0.40	
Body size + environment	4	82.51	1.68	0.08	0.48	
Colony size × body size	5	82.54	1.71	0.07	0.55	
Body size	3	82.72	1.90	0.07	0.62	
Colony size + body size + sex + condition	6	82.74	1.91	0.07	0.69	
Migration distance (gamma)						
Colony size × Body size	5	1075.11	0	0.15	0.15	1.03
Colony size	3	1075.17	0.06	0.14	0.29	
Colony size × body size × sex	9	1075.59	0.47	0.12	0.41	
Colony size + body size	4	1076.51	1.34	0.10	0.51	
Colony size + condition	4	1077.04	1.87	0.08	0.59	
Colony size + sex	4	1077.06	1.89	0.08	0.67	

**Table 3:** Model‐averaged coefficients (±*SE*) and importance values for individual covariates across the 95% confidence set of models for each movement parameter. Bold values indicate the highest importance value for each outcome

Variable	Breeding				Nonbreeding			
	50UD		95UD		Migratory strategy		Distance	
	Coefficient	Importance	Coefficient	Importance	Coefficient	Importance	Coefficient	Importance
Colony size	0.03 ± 0.01	**0.96**	0.19 ± 0.07	**0.92**	0.01 ± 0.005	0.63	0.12 ± 0.05	**0.78**
Body size (culmen)	0.7 ± 1.0	0.25	3.6 ± 6.5	0.25	−0.06 ± 0.03	**0.95**	−4.5 ± 5.5	0.34
Condition	9.3 ± 79.7	0.24	548 ± 544	0.31	−1.6 ± 2.0	0.37	−283 ± 448	0.28
Sex—Male	−10.8 ± 46.3	0.25	−43.4 ± 307	0.24	1.3 ± 1.5	0.39	−3.3 ± 278	0.28
Environment	2.3 ± 17.7	0.27	75.5 ± 120	0.29	0.2 ± 0.3	0.36	76.9 ± 86.2	0.38

A model including both colony size and body condition also received substantial support as a predictor of 95UD areas (Table 2). The relationship between body condition and 95UD area was positive, indicating an increase in 95UD area with increasing body condition. However, condition was not a significant predictor of 95UD area (*t*_65_ = 1.20, *p* > .2).

### 3.3 Chick condition and stress

**Figure 4:**
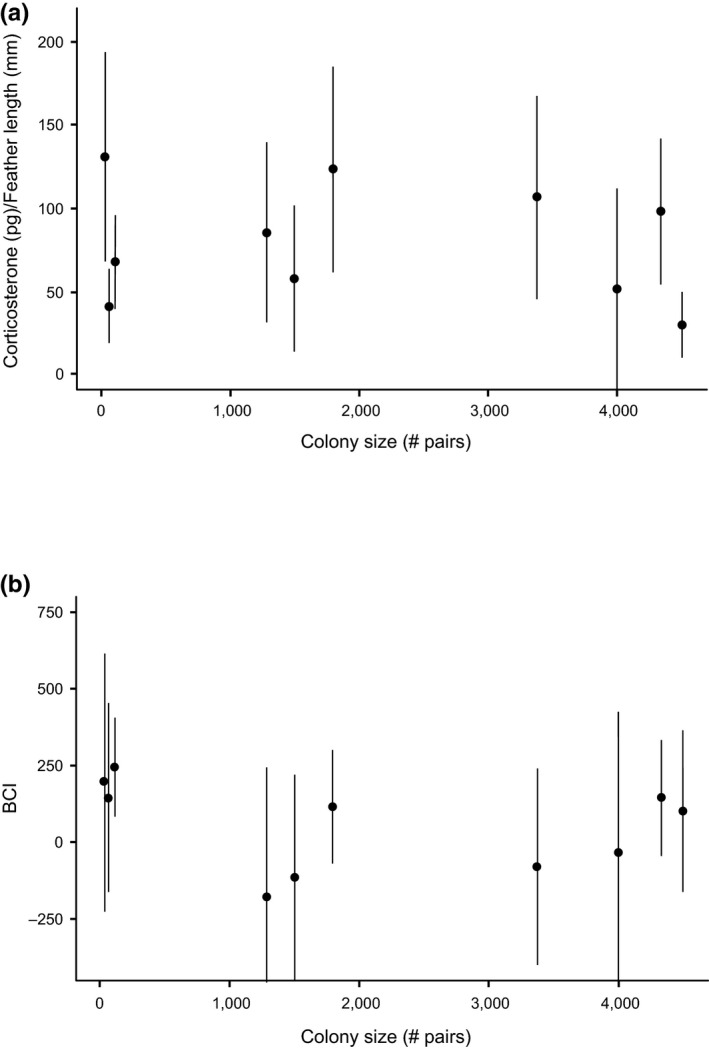
Brown pelican nestling (a) corticosterone levels, and (b) body condition index (BCI) at nine colonies in the Gulf of Mexico, 2013–2015. Colony mean values are shown ±*SD*

We found a weak negative correlation between colony size and nestling corticosterone levels (*t*_253_ = −2.00, *p* = .05) (Figure 4a). Colony size and nestling BCI were not significantly correlated (*t*_253_ = −1.04, *p* > .20) (Figure 4b). We did not find a significant relationship between environmental conditions or environment–colony size interactions and either of the chick health parameters (*p* > .20 for all variables).

### 3.4 Migratory movements

We obtained data on migratory movements of 63 individuals. Proportion of migrants per colony was similar among colony sites.

**Figure 5:**
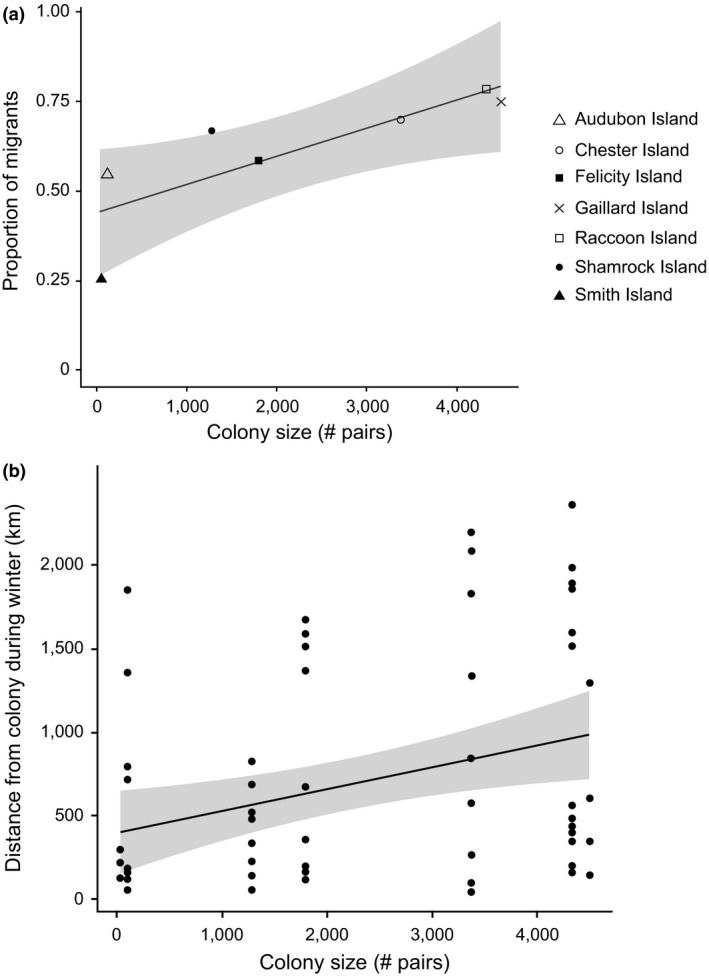
Relationships between migratory characteristics [(a) proportion of migrants; (b) average distance between summer and winter home ranges] and colony size of brown pelicans in the Gulf of Mexico, 2013–2015. Ninety five percent confidence estimates of regression lines are shaded

Colony size was included as a predictor in 11 of 13 substantially supported models of migratory movements (Table 2) and had the highest importance value among all parameters for predicting migration distance. Colony size had a significant positive correlation with both migratory strategy (*t*_62_ = 2.16, *p* = .03) and migration distance (*t*_62_ = 2.85, *p* = .006). For each increase of 100 pairs at the breeding colony, individuals were 1% more likely to migrate (Figure 5a), and wintered approximately 16 km further from their breeding sites (Figure 5b).

**Figure 6:**
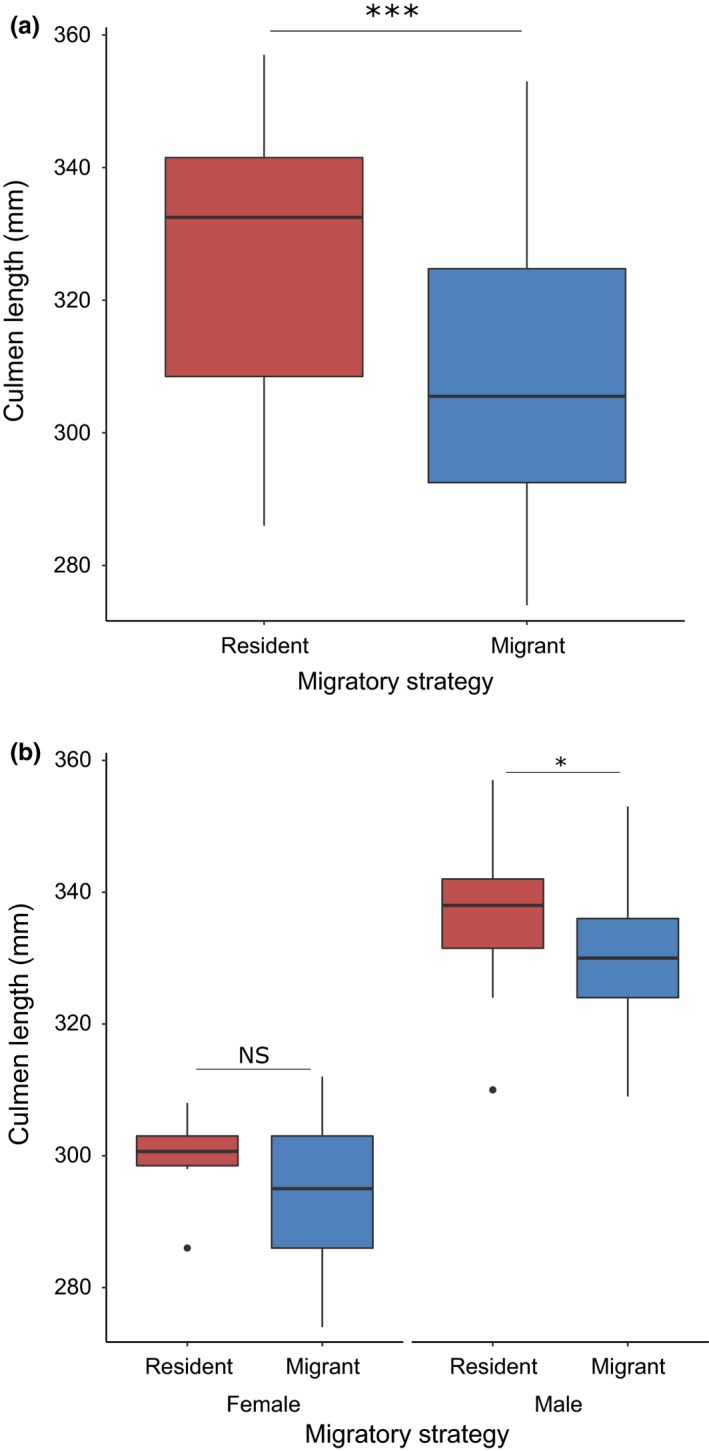
Differences in body size between resident (red) and migrant (blue) individuals for (a) all tracked brown pelicans, and (b) within sexes. Asterisks denote significance levels of between‐group differences (***p < .001; *.01 < *p* < .05; NS:* p* > .05)

Twelve of 13 top models for migratory movements included individual covariates. Body size was included in ten supported models, including all models of migratory strategy, and had the highest importance value among all parameters for predicting migratory strategy (Table 3). Body size had a negative correlation with migratory strategy (i.e., smaller individuals were more likely to migrate) (*t*_62_ = −3.15, *p* = .001; Figure 6a) but not with migration distance (*t*_62_ = −1.19, *p* > .2).

Sex was included in four of the top models. Males (47% migrants; *N* = 36) were less likely to migrate than females (78% migrants; *N* = 32) *t*_62_ = −2.50, *p* = .01), although migration distances did not differ significantly between sexes (*t*_62_ = −1.03, *p* > .2). As males were larger‐bodied than females, we also tested for within‐sex differences in body size. Resident males were significantly larger than migrant males (*F*_1,34_ = 4.65, *p* = .04), but resident and migrant females did not differ significantly in body size (*F*_1,30_ = 2.18, *p* = .14) (Figure 6b).

Body condition was included in three of the top models, and environmental parameters appeared in one model, but neither was a significant predictor of either migratory strategy or migration distance (*p* > .2 in all cases).

Interaction terms were included in three of the 13 supported models (Table 2). A model including colony size, body size, and their interaction was the best‐supported model of migration distance and was among the top models of migration strategy. A model including colony size, body size, sex, and their interactions was included among the top models of migration distance.

## 4 DISCUSSION

Density‐dependent competition for prey resources is one of several factors potentially influencing breeding ecology, foraging distances, and migratory movements of colonial seabirds. The goal of our study was to test for effects of density‐dependent resource competition on several parameters related to movement patterns and reproductive success in brown pelicans, and to relate these effects to reproductive output and between‐individual variation.

While we chose study colonies within a single ecoregion to limit environmental variation, we were unable to control all factors that could contribute to local variation in foraging conditions. Underlying resource availability, which is difficult to measure directly in marine systems, may also vary between colonies and hence confound an assessment of the influence of colony size on seabird behavior. Gulf menhaden (*Brevoortia patronus*), which comprise a large portion of pelican diets in the Northern Gulf of Mexico (Shields, 2014), are concentrated in the central portion of the Gulf, meaning that colonies at the margins of our study area were at the edges of the range of Gulf menhaden and therefore may have experienced lower availability of this prey item. To help account for this underlying variation, we incorporated remotely sensed environmental variables associated with menhaden availability (Ahrenholz, 1991; Christmas et al., 1982) into our models of adult movement patterns and chick condition. However, such variables are only a proxy for underlying prey variation, and the most effective way to account for prey availability would be to measure prey concentrations directly.

In this study, we addressed three principal predictions related to the operation of density‐dependent prey depletion:

### 4.1 Prediction 1: Individuals nesting in larger breeding colonies will travel greater distances to forage during breeding

We found a strong linear increase in the size of both core and full home ranges of individual breeders with the size of the breeding colony, meaning that individuals at larger colonies consistently foraged over larger areas than individuals at smaller colonies. Body condition was included as a predictor in one of our top models of full home range area, but only in combination with colony size, and breeders that foraged over greater distances were generally in better physical condition. Other individual characteristics (sex, body size) and regional environmental conditions were not included as predictors in any highly supported models of either core or full home range areas during the breeding season. This adds to a growing body of evidence that colonial birds consistently increase their foraging radius in response to localized density‐dependent prey depletion (e.g., Ainley, Ford, Brown, Suryan, & Irons, 2003; Bonal & Aparicio, 2008; Brown & Brown, 1996; Elliott et al., 2009; Ford et al., 2007; Lewis et al., 2001). The fact that we did not observe a decline in adult body condition with increased foraging area further suggests that pelicans in this system were able to increase their foraging effort without experiencing compromised physical condition.

As most research to date has concentrated on pelagic seabirds breeding at temperate latitudes, our study adds a new perspective to the understanding of the relationship between colony size and foraging distance in seabirds. For instance, in contrast to previous studies (e.g., Grémillet et al., 2004; Wakefield et al., 2013), we did not observe strong spatial segregation in foraging ranges between closely neighboring colonies. For example, adults from two Florida colonies (Audubon and Smith Islands) frequently traveled over 100 km to a common foraging area at the mouth of the Apalachicola River. Prey concentrations in nearshore environments may occur predictably in and around stationary coastal features including headlands, river mouths, and upwelling zones (Becker & Beissinger, 2003). Thus, the overlap we observe between neighboring colonies may represent common exploitation of prey‐concentrating features that are spatially predictable.

### 4.2 Prediction 2: Individuals nesting in larger breeding colonies will raise poorer‐quality nestlings

We did not find a significant relationship between colony size and either of the nestling condition metrics we tested (body condition or feather corticosterone). We have previously determined that both feather corticosterone and body condition are effective predictors of chick survival in this system (Lamb et al., 2016), so we can extrapolate from our results that the reproductive rates of pelicans do not decline with colony size. This result contradicts several previous studies suggesting a relationship between chick condition and colony size (e.g., Cairns, 1992; Gaston et al., 1983; Hunt et al., 1986); however, several other studies have failed to find a correlation (Ainley et al., 2004; Brown & Brown, 1996; Gaston et al., 2007). The fact that we found a relationship of colony size to adult foraging ranges, but not chick condition, indicates that, within the range of colony sizes included in this study, adults can adjust their foraging ranges in response to density‐dependent prey depletion without sacrificing reproductive output. Pelicans in this system may be operating well below metabolic limitations on their energetic expenditure (Drent & Daan, 1980), and thus be capable of plasticity in foraging effort.

This study also differs from previous studies of the effects of colony size on seabird breeding success by focusing on nearshore seabirds in subtropical waters, while previous studies that have suggested a negative effect of colony size on chick condition have been conducted in high‐latitude, pelagic systems. Both the life history strategies of nearshore compared to pelagic seabirds and the relative complexity of nearshore compared to pelagic habitats may affect the relationship between colony size and chick condition (Suryan, Irons, Brown, Jodice, & Roby, 2006). For example, nearshore seabirds tend to have a more variable clutch and brood size compared to pelagic seabirds, allowing for adjustments in reproductive output in response to changes in local prey availability. Similarly, higher concentrations of resources within nearshore environments compared to pelagic habitats may allow nearshore seabirds to remain well below their energetic thresholds during chick‐rearing (Ballance et al., 2009); thus, increases in foraging effort due to density‐dependent competition might be less likely to result in measurable declines in chick condition than in pelagic environments.

### 4.3 Prediction 3: Individuals nesting in larger breeding colonies will be more likely to migrate and will travel farther from the colony during nonbreeding

We found a positive correlation between breeding colony size and the proportion of individuals that migrated away from the colony during nonbreeding, as well as the distance traveled by migrants. Partial migration in seabirds has been little‐studied and, to the best of our knowledge, a relationship between migratory strategies of individual breeders and breeding colony size has not previously been observed in either nearshore or pelagic seabirds. Density‐dependent competition for resources may present a significant obstacle to remaining resident in the subtropical northern Gulf of Mexico. During winter months, prey populations in the region migrate offshore, and shallow waters may freeze during periods of extreme cold and further reduce availability of prey (Christmas et al., 1982). By reducing predation pressure during periods of resource scarcity, partial migration provides a potential mechanism for increasing overwinter survival in the face of density‐dependent competition.

Previous research on density‐dependent population regulation in seabirds has focused almost exclusively on foraging movements and nesting health during the breeding season. The study of migratory behavior in relation to conspecific prey depletion due to density dependence has been less common and has primarily been limited to species‐level patterns (Diamond, 1978). In contrast, investigations of relationships between colony size and migratory behavior within a single species have been rare. Previous evidence has indicated a complex migration strategy in brown pelicans (King et al., 2013), but has not explored how migratory behavior varies throughout the population or what drives individual migration patterns.

In addition to suggesting a relationship between colony size and migration propensity, our results also highlight the importance of individual physical characteristics in driving migration patterns. Whether individuals were migratory or resident was highly dependent on body size, as well as the interaction between body size and colony size. Five of the seven top models of migratory strategy included both body size and colony size, including one model with an interaction between the two covariates. The best‐supported model of migration distance included a body size–colony size interaction. Partial migration patterns have previously been associated with individual differences in social status (e.g., Cristol, Baker, & Carbone, 1999; Terrill, 1987), variation in thermal tolerance with body size (e.g., Belthoff & Gauthreaux, 1991; Chapman et al., 2011; Macdonald, McKinnon, Gilchrist, & Love, 2016), or differential fitness benefits to males of early arrival at the breeding site (e.g., Myers, 1981; Pérez et al., 2013). The majority of our top models for migratory behavior contained colony size in combination or interaction with one or more individual characteristics (sex, body size, and/or condition), indicating that the influence of individual characteristics on migration propensity and distance is mediated by density‐dependent competition. Smaller individuals and females were more likely to migrate overall and were increasingly likely to migrate as colony size increased, lending support to the importance of social status as a driver of migration decisions. Local intraspecific competition may place subdominant individuals at a competitive disadvantage during periods of reduced prey availability and force them to move further from colony sites during the winter.

Our results offer insight into the ecological underpinnings of migratory decisions, suggesting that local intraspecific competition may be a driver of partial migration, and that changes to brown pelican breeding densities could result in corresponding shifts in migratory behavior and nonbreeding locations that differentially affect individuals within the population. The relationship between colony size and migration includes a complex combination of factors including competition, survival, and site selection. By establishing a link between intraspecific competition and migration, our results may elucidate a demographic mechanism underlying the differences observed in migration strategies among individuals.

## CONFLICT OF INTEREST

None declared.

## DATA ACCESSIBILITY

The data used in this study are available on Movebank (movebank.org, Movebank Study ID 296027617) and are published in the Movebank Data Repository with DOI 10.5441/001/1.7856r086.
