# Supplementary material for: Influence of density‐dependent competition on foraging and migratory behavior of a subtropical colonial seabird
Source: Ecol Evol. 2017 Jul 10;7(16):6469–81. doi: 10.1002/ece3.3216 (PMC5574757; doi:10.1002/ece3.3216)
Supplement: Supplementary file 1 [file ECE3-7-6469-s001.docx]

Table S1: Sources and resolutions of remotely-sensed environmental variables used to calculate local environmental conditions at colony sites.

| Variable name | Layer name | Data source | Resolution |
| --- | --- | --- | --- |
| Bathymetry | 2-minute Gridded Global Relief Data, (ETOPO2) v2 | NOAA National Geophysical Data Center | 0.033 |
| Bottom substrate | Dominant Bottom Types and Habitats | Gulf of Mexico Data Atlas, NOAA | 0.1 |
| Net primary production | Vertically Generated Production Model | Ocean Productivity Database, Oregon State University | 0.083 |
| Sea surface temperature | Sea Surface Temperature, Climatological Mean, 10 m depth | NOAA National Centers for Environmental Information (Boyer et al. 2011) | 0.1 |
| Sea surface salinity | Sea Surface Salinity, Climatological Mean, 10 m depth | NOAA National Centers for Environmental Information (Boyer et al. 2011) | 0.1 |
